# Clinical Lectures on Diseases Peculiar to Women

**Published:** 1879-04

**Authors:** 


Clinical Lectures on Diseases Peculiar to Women. By Lombe Atthill, M D., University of Dublin, Master of the Rotunda Hospital, etc. Fifth Edition, revised and enlarged, with Illustrations. Philadelphia: Lindsay & Blakiston. 1879.

The number of editions this work has gone through indicates the estimation placed upon it by the profession. While the subjects are treated of in a succinct manner, it lacks nothing in a practical and scientific exposition of this branch of medicine.

The author has well succeeded in "furnishing to practitioners and students 'within the limits of a moderate-sized volume such an account of the diseases peculiar to women, verified by his personal experience, as would meet their wants.'"

It is not necessary that we should review in detail each operation and disease treated of in this valuable volume; suffice it to say that the pathology and treatment, medical and surgical, of uterine and ovarian disease, is discussed *in extenso*, and presented in such form as to make it a most valuable book to both practitioner and student. The volume is gotten up in good style and reflects no little credit on the publishers, Messrs. Lindsay & Blakiston, Philadelphia.

